# Clarifying the Evidence Base for “Personalized Learning Path Design” Claims in Peng et al. (2025)—A Letter to the Editor

**DOI:** 10.1002/nop2.70510

**Published:** 2026-03-27

**Authors:** San‐Ping Wang, Yun‐Ling Liu

**Affiliations:** ^1^ Taoyuan Psychiatric Center Ministry of Health and Welfare Taoyuan Taiwan; ^2^ Department of Occupational Therapy, College of Medicine Chang Gung University Taoyuan Taiwan

Dear Editor,

We read Peng et al. (2025), “ChatGPT Applications in Nursing: Current Status and Future Perspectives,” with great interest. The paper offers a timely and well‐organized synthesis of ChatGPT's emerging roles across clinical support, nurse education, and patient services, and we appreciate its balanced discussion of technical and ethical challenges. The overview of clinical documentation efficiency gains and risk‐aware adoption is especially useful for nursing stakeholders considering early implementations.

We write, however, to request clarification regarding two contiguous sentences in Section 2.2 Nurse Education Training:

Personalized learning path design highlights ChatGPT's data‐driven advantages. Through machine learning to analyse students' knowledge graph gaps and skill mastery curves, ChatGPT can dynamically adjust the difficulty gradient and presentation of teaching content.

Our understanding is that these sentences appear as narrative assertions without an immediate parenthetical citation; the next sentence cites Chang et al. 2024 as quasi‐experimental evidence for curriculum‐level benefits after integrating a ChatGPT system (improvements in problem‐solving, critical thinking, and learning enjoyment). While valuable, *Chang et al*. does not, to our reading, implement or evaluate mechanisms such as knowledge‐graph gap detection, mastery‐curve estimation, or automated difficulty adaptation at the level described in the quoted sentences. Thus, the cited study seems to support *educational outcomes of integration*, rather than *the mechanism* of adaptive personalization claimed just prior. We would be grateful if the authors could clarify whether there are primary sources demonstrating a ChatGPT‐based (or ChatGPT‐integrated) system that: (a) infers learners' knowledge‐graph gaps, (b) estimates skill/mastery trajectories, and (c) dynamically adapts content difficulty accordingly. If so, could those be cited directly at the claim location?

Relatedly, the phrase “ChatGPT's data‐driven advantages” is admittedly broad. If the intention was to describe LLM‐assisted teaching tools that *integrate* external learning analytics (e.g., item‐response models, Bayesian/DKT knowledge tracing, or platform‐level learner telemetry) to drive adaptation—rather than the base ChatGPT model alone—stating this integration explicitly would help delimit scope and avoid over‐attribution to ChatGPT per se. Such precision would also align with the paper's otherwise careful articulation of limitations (e.g., model hallucinations, knowledge‐update lag, and multimodal constraints), which we commend.

To strengthen this useful section, we respectfully suggest the following options:

1. Cite mechanism‐level evidence (if available): Add primary studies that *demonstrate* knowledge‐graph gap analysis and mastery‐curve–driven adaptation in nursing or health‐education contexts using ChatGPT or ChatGPT‐integrated systems; place these citations immediately after the two mechanism sentences.
2. Qualify as future‐facing potential (if such evidence is not yet available): Rephrase to indicate that *with integration into learning‐analytics infrastructures*, ChatGPT‐based assistants could support adaptive pathways; then keep Chang et al. 2024 as evidence for *course‐level benefits*, not mechanism verification. This would mirror the paper's forward‐looking tone in other parts (e.g., multimodal evolution and standards work).
3. Briefly delineate system architecture (one to two clauses): Specify the data sources (e.g., quiz logs, assignment telemetry), the analytic layer (e.g., IRT/BKT/DKT), and the adaptation targets (difficulty, format, scaffolding). Such detail will help readers distinguish between LLM‐mediated tutoring and analytics‐driven adaptive learning in nursing education.

We reiterate our overall support for the paper's contribution. Clarifying the evidentiary basis and scope of the “personalized learning path design” statements will, in our view, make an already valuable narrative review even more actionable for nurse educators and curriculum designers.

## Funding

The authors have nothing to report.

## Conflicts of Interest

The authors declare no conflicts of interest.

## Data Availability

Data sharing not applicable to this article as no datasets were generated or analysed during the current study.
